# Body composition and prediction equations using skinfold thickness for body fat percentage in Southern Brazilian adolescents

**DOI:** 10.1371/journal.pone.0184854

**Published:** 2017-09-14

**Authors:** Wagner Luis Ripka, Leandra Ulbricht, Pedro Miguel Gewehr

**Affiliations:** 1 Graduate Program in Electrical and Computer Engineering, Federal University of Technology—Paraná, Curitiba, Brazil; 2 Graduate Program in Biomedical Engineering, Federal University of Technology—Paraná, Curitiba, Brazil; Medizinische Universitat Innsbruck, AUSTRIA

## Abstract

**Objectives:**

The purpose of this study was to: a) determine the nutritional status of Brazilian adolescents, and; b) present a skinfold thickness model (ST) to estimate body fat developed with Brazilian samples, using dual energy x-ray absorptiometry (DXA) as reference method.

**Methods:**

The main study group was composed of 374 adolescents, and further 42 adolescents for the validation group. Weight, height, waist circumference measurements, and body mass index (BMI) were collected, as well as nine ST–biceps (BI), triceps (TR), chest (CH), axillary (AX) subscapularis (SB), abdominal (AB), suprailiac (SI), medial thigh (TH), calf (CF), and fat percentage (%BF) obtained by DXA.

**Results:**

The prevalence of overweight in adolescents was 20.9%, and obesity 5.8%. Regression analysis through ordinary least square method (OLS) allowed obtainment of three equations with values of R^2^ = 0.935, 0.912 and 0.850, standard error estimated = 1.79, 1.78 and 1.87, and bias = 0.06, 0.20 and 0.05, respectively.

**Conclusion:**

the innovation of this study lies in presenting new regression equations for predicting body fat in Southern Brazilian adolescents based on a representative and heterogeneous sample from DXA.

## Introduction

The increasing number of children and adolescents with excess body fat represents a potential risk to this population’s health, considering it is directly related to hypertension, respiratory problems and the development of psychosocial complications [1, 2]. An epidemiological study carried out in 2010, involving 144 countries, found that overweight prevalence and infant obesity in the world was 6.7%, and estimated an increase of 35% by 2020, with developing countries being the most affected [3].

In countries which represent areas with high risk of overweight and infant obesity such as Brazil [3], where more than 70% of the population utilize the public health system [4], monitoring body composition and developing procedures that approach reality in relation to body fat quantification are essential to diagnose and prevent infant obesity–a condition that the World Health Organization (WHO) describes as a worldwide epidemic [5].

Among various changes in evaluation forms used to assess body composition, the body mass index (BMI) is the most common instrument and, despite its low cost and convenience, it is limited in relation to distinction fat mass and muscle mass, though it is recommended as a general evaluation tool to investigate the population’s nutritional status [6]. Another method based on low cost anthropometric measurements is waist circumference (WC), which presents high sensitivity and specificity to central fat evaluation in children and adolescents [7]. Similarly to BMI, WC provides only a general overview of body composition. At another extreme, dual energy X-ray absorptiometry (DXA), provides a high-precision technique capable of quantifying body fat. Recognized as a reference method to body composition analysis, DXA has a high cost and needs laboratory evaluation, such factors represent its main limitations [8, 9].

An inexpensive instrument which overcomes BMI limitations, preserving a high correlation with DXA method, is subcutaneous fat evaluation by skinfold thickness (ST). ST techniques have their use based on equations developed from mathematical regressions to estimate body composition in different ethnic and age groups. Additionally, proper choice of the equation is essential to the correct assessment of children and adolescents’ body composition [10, 11].

There is no knowledge about ST-based equations, developed from a sample that meets the adolescent range (12–17 years) in Brazil. This fact induces the use of models based on foreign samples, a practice that has experienced high discrepancies in the evaluation and diagnosis of adolescents’ nutritional status [11, 12].

Thus, this study aims to: a) determine the nutritional status of Brazilian adolescents, and; b) present an ST model to estimate body fat developed with Southern Brazilian sample, using DXA as the reference method.

## Material and methods

### Target population

The population was formed by adolescents (12–17) enrolled in the school system between 2015–2016. The sample was determined from sampling error of 4% over confidence level of 95% from the adolescent population.

This study was submitted to ethical evaluation via Brazil Platform, being approved under the number: 11583113.7.0000.5547. Parents gave permission by signing a consent form. The exclusion criteria considered adolescents: a) whose parents did not allow participation; b) who made use of medicines containing calcium; c) who were examined with radiography seven days before the evaluation.

### Anthropometric assessment

All anthropometric measurements were followed by the obtainment of body mass, height, WC, ST measurements and evaluation by DXA.

Body mass was measured with an analog mechanical scale (Filizola—São Paulo, Brazil), with capacity for up to 160kg, and 100g of resolution. The assessed sample had to wear shorts only. For height measurement, a stadiometer attached to a scale was used, with 0.1 cm resolution.

For the circumference values, a flexible anthropometric measuring tape of 2m, graduated in mm, was used (Cardiomed—Curitiba, Brazil). Waist circumference was defined at the minimum circumference between the iliac crest and the rib cage.

Two appraisers, duly trained, were responsible for collecting ST and had as technical error 3.5% and 3.2%. It is noteworthy that for the ST measures, values lower than 5% (intra-rater) are considered good [13]. For this evaluation, a scientific ST caliper, Cescorf (Porto Alegre, Brazil) was used. Skinfold caliper has a constant compression equal 10 g.mm^-2^. Measures were collected in mm from nine anatomical sites, on right side of body: biceps (BI), triceps (TR), chest (CH), axillary (AX), subscapularis (SB), abdominal (AB), suprailiac (SI) medial thigh (TH), and calf (CF). Anatomy landmarks followed guidelines proposed by the International Society for the Advancement of Kinanthropometry [14]. Duplicate measurements were performed at all points. If there was 2mm difference, a third collection was performed. At the end, the measurements arithmetic average to determine the anatomical point value was taken.

Evaluation with DXA was performed using a Hologic QDR Discovery scanner fan-beam, scanning type (Hologic, Inc., Bedford, MA, USA). In this evaluation, individuals were positioned in dorsal decubitus on the scanner bed. The presence of metal artifacts was not allowed, such as earrings, chains or rings, as well as clothes containing any kind of metal. Total fat percentage (%BF) was obtained automatically by the machine’s software.

### Data analysis

**Fig 1:**
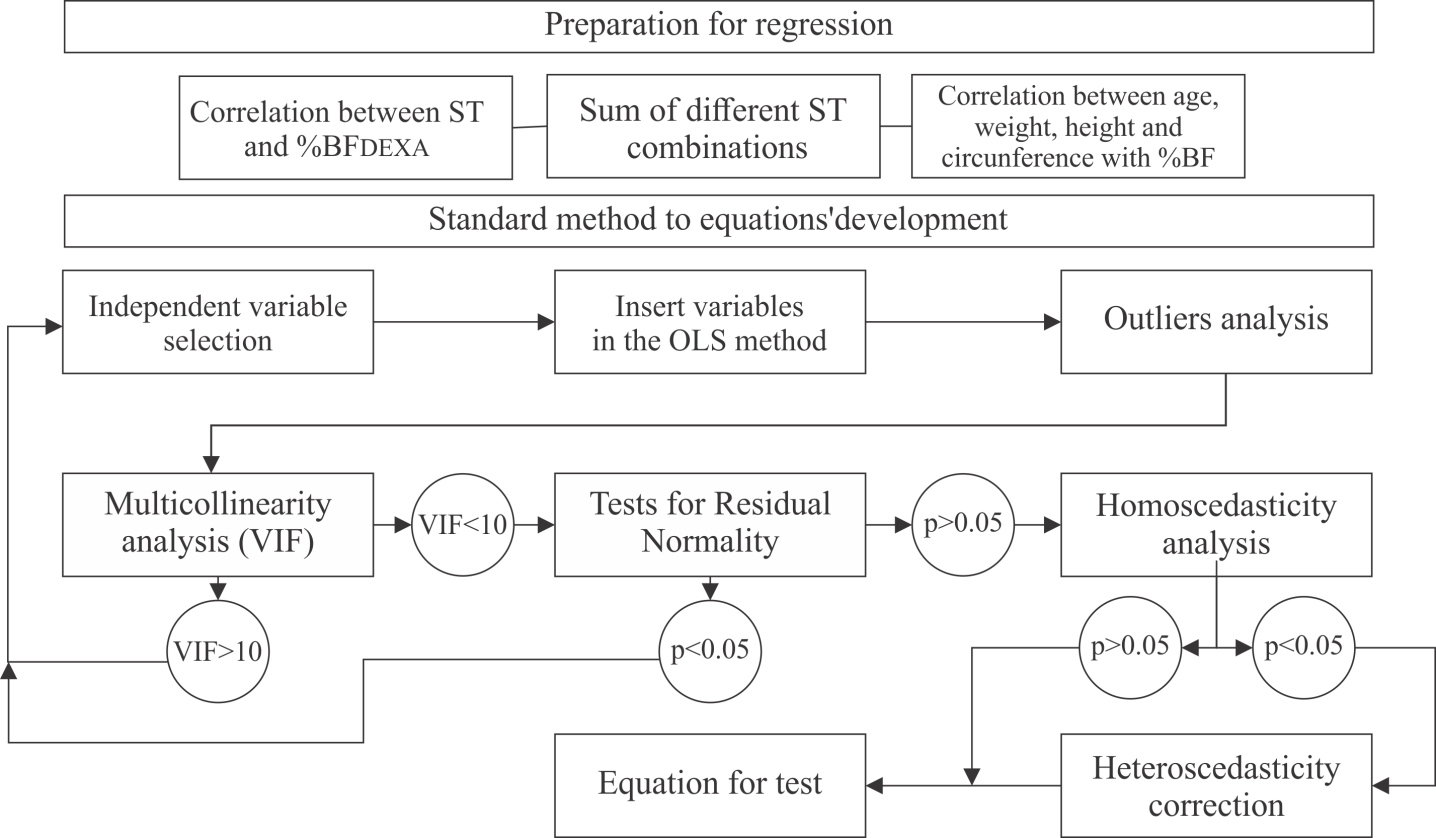
Flow chart of the equations assembling process to predict body fat percentage (%BF). Where: skinfold thickness (ST); ordinary least square (OLS); variance inflation factor (VIF).

Average values and standard deviation data were presented to the group and segmented by chronological age. For the equations development, we have used a multiple linear regression model through the ordinary least square method (OLS) [15]. The %BF obtained through DXA was used as a dependent variable in the models. Height, weight, age, ST, and WC were utilized as independent variables. As for ST measures, they were accepted whether individually or added up. For inclusion or exclusion of independent variables, and for development of %BF prediction models, the steps presented in Fig 1 were followed.

The parametric bivariate analysis of the variables association was performed with the application of the Pearson correlation coefficient. For the analysis and outlier removal, a test from studentized residuals was applied. The outliers were defined as values higher than 1.5 times the interquartile range + 3^rd^ quartile (AIQ), and values 1.5 times lower than 1^st^ quartile–AIQ, where AIQ is defined as the difference between the 1^st^ and 3^rd^ quartiles. For the multicollinearity analysis we appealed to the Variance Inflation Factor (VIF), where variables with values greater than 10 were considered estimation problems and were removed from the model [16]. The normal analysis was carried out by using the Shapiro-Wilk test. The homoscedasticity analysis of regression models residues were made by White statistical test [17]. The study also used Bland-Altman graphical analysis to determined concordance between equations developed with the DXA method.

Finally, as selection criteria, equations that presented the following were chosen: a) lower standard estimate error (SEE); b) higher value of determination coefficient (r^2^), and;c) fewer independent variables.

The validation of equations was performed in a sample originally removed from the total population of the study. Such sample population was not considered in the equation development. Individuals were randomly selected in all studied age groups. Total Error values (TE) and SEE were calculated according to Lohman [18] recommendations. Likewise, the average difference between DXA and the equations results was verified using paired samples test.

To calculate BMI values (kg/m^2^) the classification curves proposed by the WHO with analysis based on age and sex were used.

To perform the analysis, statistical significance p value was<0.05. All tests were performed using the software Statistical Package for Social Sciences (SPSS), 17.0 version (SPSS Inc. Chicago, IL) and GNU Regression software, econometrics and time-series library (GRETL), 2016 version.

## Results

Three hundred and seventy four adolescents with average age of 14.85±1.61 years were assessed, divided by age groups. Table 1 discloses descriptive values to the anthropometric and DXA variables. A progressive increase in weight, height and WC variables with age is observed. Age groups of 12 and 16 years old were the ones that presented the highest and lowest %BF (27.69±7.49% and 18.06±4.31%), respectively.

**Table 1:** Descriptive values (average ± SD) for anthropometric measurements and adolescents’ body composition.

Age (y)	n	Weight (kg)	Height (m)	BMI (kg/m^2^)	WC (cm)	DXA Variables	
						Body Fat (%)	FM (kg)
12	39	47.54±13.21	1.51±0.08	20.54±4.94	67.91±9.59	27.69±7.49	14.54±6.99
13	41	52.33±11.38	1.61±0.08	20.03±3.21	68.86±8.55	23.75±5.69	13.01±5.56
14	80	59.22±13.24	1.67±0.08	21.02±3.61	70.08±7.67	20.02±4.85	12.75±6.93
15	67	61.81±8.97	1.71±0.05	21.09±2.49	70.97±5.61	19.33±4.26	13.24±6.78
16	72	63.67±9.44	1.73±0.07	21.10±2.70	71.44±6.11	18.06±4.31	12.32±4.70
17	75	68.78±10.19	1.75±0.06	23.09±6.50	75.24±7.45	19.51±4.83	14.83±8.34
Total	374	60.48±12.72	1.68±0.10	21.30±4.26	71.20±7.65	20.63±5.81	13.41±6.78

Where: Waist Circumference (WC); Body fat percentage (%BF); Fat mass (FM); Body Mass Index (BMI).

Regarding the nutritional status classification by BMI the percentile curve, results found an overweight prevalence in 20.3% (n = 76) of the individuals, and obesity in 5.6% (n = 21).

As for the ST analysis, thigh (r = 0.880), triceps (r = 0.874), calf (r = 0.859), and biceps (r = 0.850) were the anatomical points of highest correlation with the % BF variable, since subscapularis measurements (r = 0.784) and medium axillary (r = 0.800) reported the lowest correlation between ST (Table 2).

**Table 2:** Correlation values with age, anthropometric variables, body fat percentage (%BF) and fat mass (FM) in male adolescents.

Variables	%BF	FM (kg)
Age (years)	-0.403*	0.027
Height (m)	-0.322*	0.174*
Waist Circunference (cm)	0.401*	0.599*
Thigh (mm)	0.880*	0.571*
Medium Axillary (mm)	0.800*	0.612*
Chest (mm)	0.840*	0.560*
Biceps (mm)	0.850*	0.533*
Calf (mm)	0.859*	0.557*
Subscapularis (mm)	0.784*	0.626*
Abdominal (mm)	0.831*	0.652*
Suprailiac (mm)	0.821*	0.627*
Triceps (mm)	0.874*	0.582*

* significant Pearson correlation coefficient (p<0.05).

High correlation between ST independent variables, verified in Table 2, is a warning sign of multicollinearity problem. Therefore, for the production of tests of a generalized equation we applied the combination of anatomical points to create new independent variables. Thus, 216 different combinations were produced.

Regression analysis from OLS method, used to obtain a parsimonious model which made possible predicting %BF, enabled the development of three equations with normal waste distribution and without heteroscedasticity. Table 3 reports the equations model with four, three and two variables to predict body fat with their respective values of r^2^, SEE, bias and limits of agreement obtained by Bland-Altman analysis.

**Table 3:** Equations to predict fat percentage in adolescents based in skinfold thickness (12–17 years old).

	Models	R^2^	SEE	BIAS	Limits of Agreement	p
EQ1	%BF predicted = 25.20–0.19(age)-6.70(height)+0.21(∑ TH, TR, BI, SB)	0.935	1.79	0.06	-3.43 to 3.31	0.361
EQ2	%BF predicted = 25.57–0.22(age)-6.69(height)+0.28(∑ TR, CF, SB)	0.912	1.78	0.20	-3.75 to 3.35	0.633
EQ3	%BF predicted = 33.60–0.50(age)-8.47(height)+0.38(∑ TR, SB)	0.850	1.87	0.05	-3.97 to 3.87	0.255

Where: equation (EQ); triceps (TR); subescapularis (SB); thigh (TH); calf (CF); biceps (BI); significant Pearson correlation p<0,05 (*); standard error estimate (SEE); body fat percentage (%BF).

**Fig 2:**
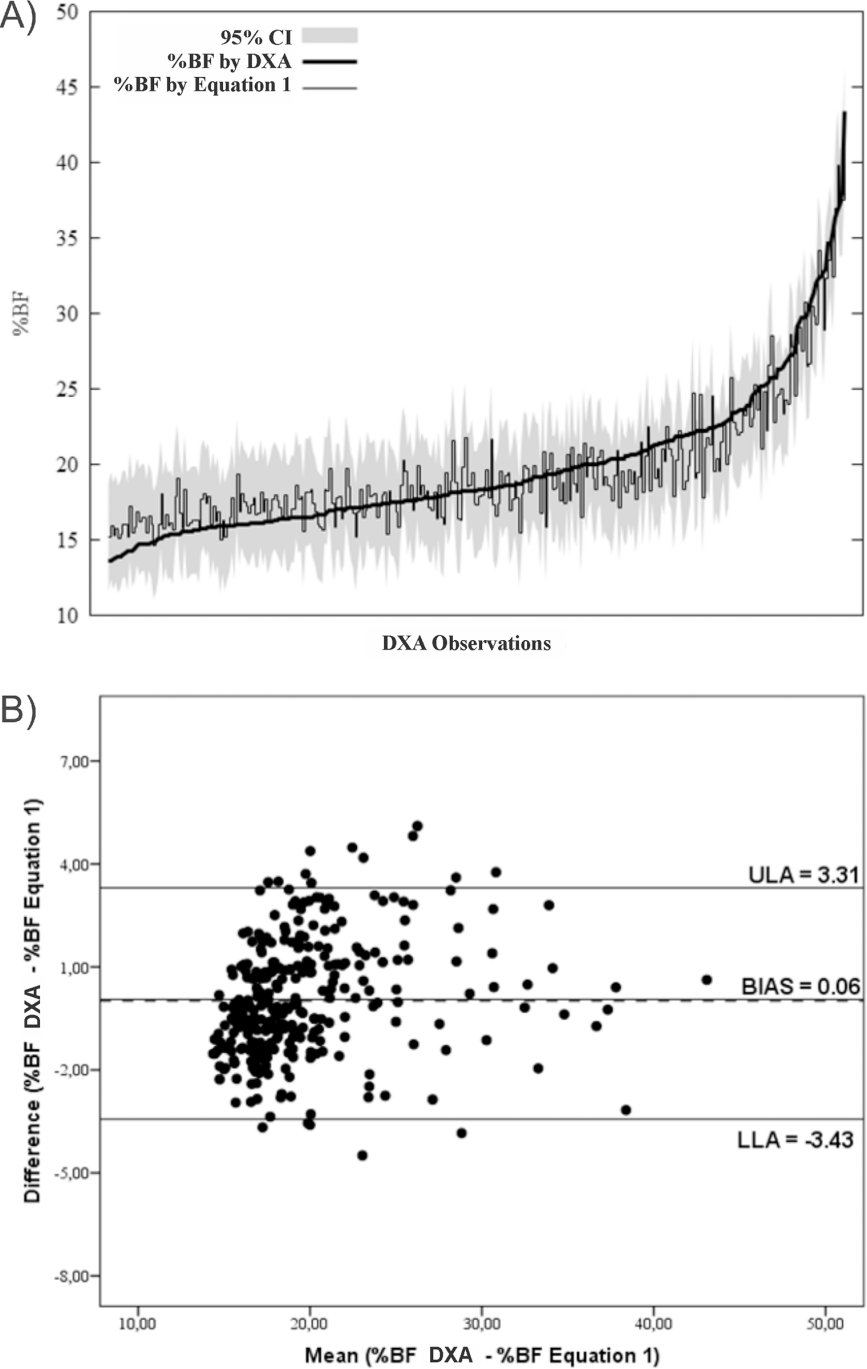
(A) Prediction analysis and tolerance limits of %BF predictive values of Equation 1 with % BF and DXA values; (B) Bland-Altman analysis of agreement %BF by DXA -%BF by Equation 1.

**Fig 3:**
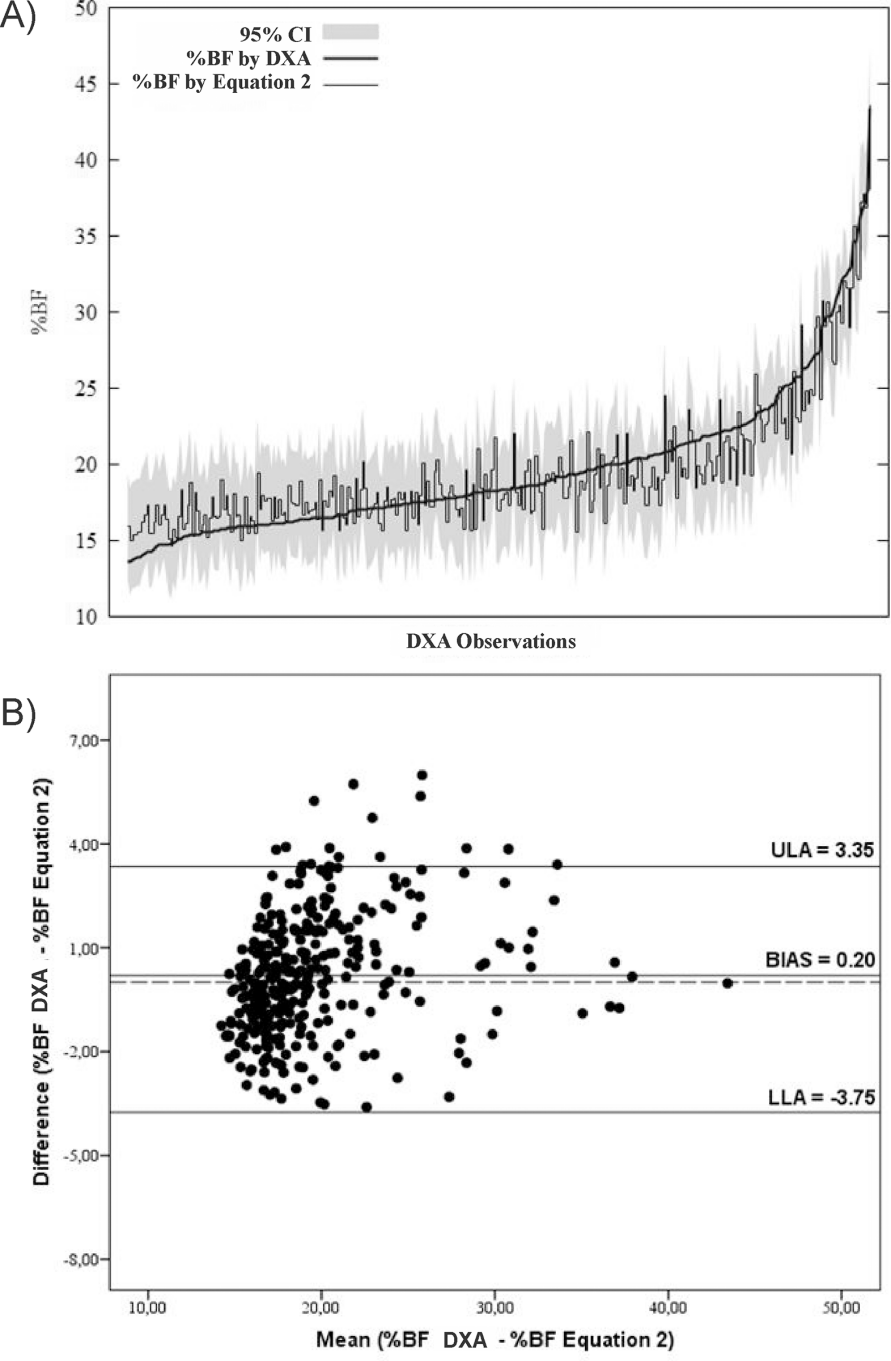
(A) Prediction analysis and tolerance limits of %BF predictive values of Equation 2 with % BF and DXA values; (B) Bland-Altman analysis of agreement %BF by DXA -%BF by Equation 2.

**Fig 4:**
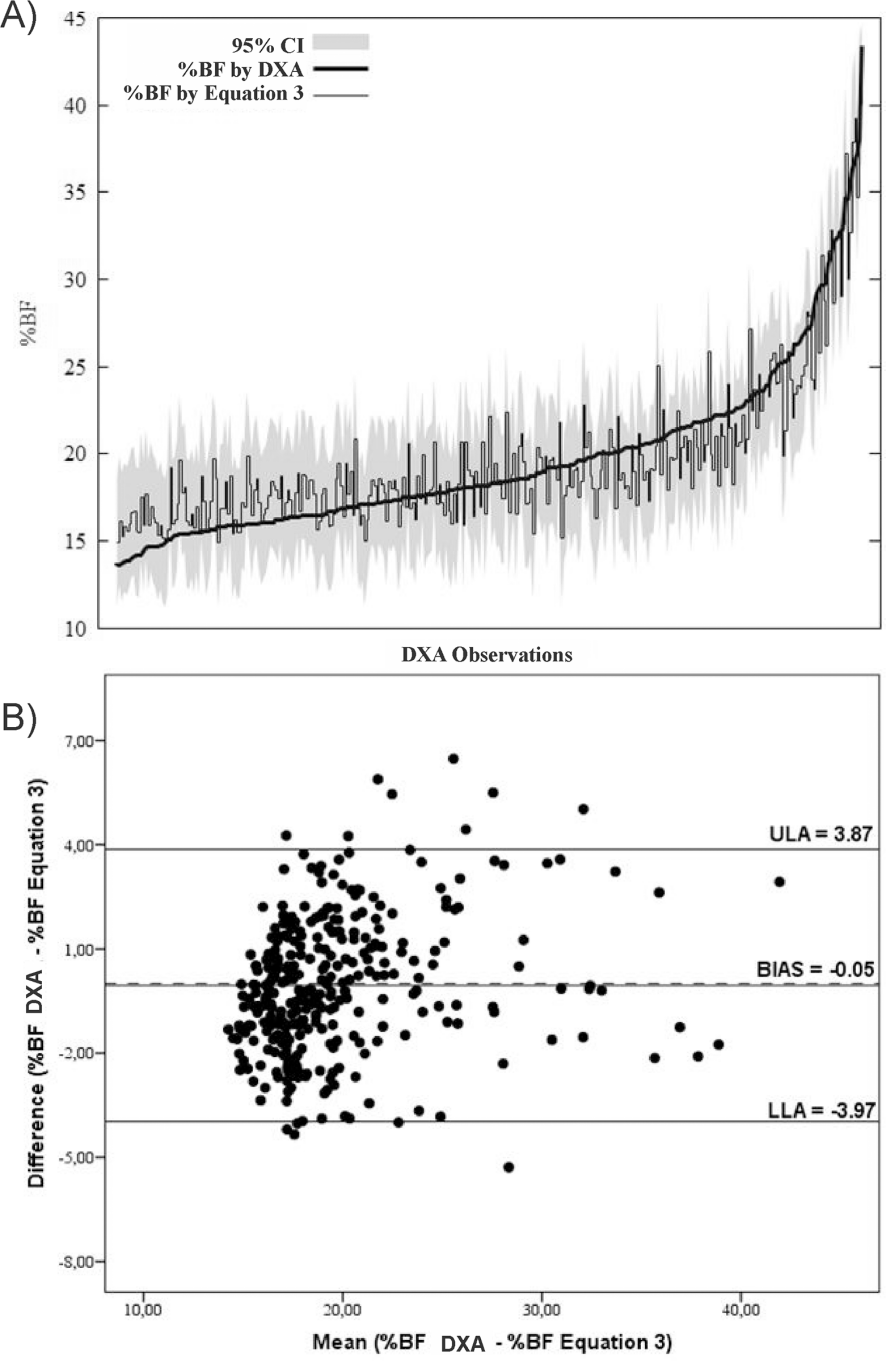
(A) Prediction analysis and tolerance limits of %BF predictive values of Equation 3 with % BF and DXA values; (B) Bland-Altman analysis of agreement %BF by DXA -%BF by Equation 3.

Figs 2–4 submit predictive analysis of % BF values compared to DXA values, as well as graphical analysis of agreement between methods. It is possible to note that equations, as compared to DXA values, seem to underestimate %BF values to obese individuals and overestimate the values for slimmer individuals.

One aspect in the development of new models for estimating body composition measures is their validation in an independent sample from that used in their development. Hence, in order to validate the equations a sample of 42 adolescents with average age 14.83±1.59 years was used. The descriptive characteristics of the sample are presented in S1 File. Table 4 portrays validation values for the three proposed models. Furthermore, S1–S3 Figs showed the Bland-Altman plots with the validation sample.

**Table 4:** Validation of equations developed in an independent sample (n = 42).

Model	Mean±SD	*R*	t	p	SEE	TE	BIAS*	Limits of Agreement*
EQ1	19,93±5.04	0.874	-0.822	0.416	2.45	0.67	0.44	-0.09 to 0.97
EQ2	19,88±5.16	0.836	-0.830	0.411	2.83	0.71	0.48	-1.44 to 2.39
EQ3	19,98±4.90	0.827	-0.597	0.554	2.75	0.63	0.11	-4.36 to 4.15
DXA	20,63±5.81							

Where: equation (EQ); standard deviation (SD); standard error estimate (SEE); body fat percentage (%BF); Total error (TE); Bland-Altman analysis (*).

Despite showing lower values of %BF as compared to DXA, the models showed no significant difference from the reference method. However, we noted that a smaller number of skinfold thickness sites as independent variables provides worst limits of agreement.

## Discussion

As its first goal, the study evaluated changes in the nutritional status of Brazilian adolescents aged 12–17 years old from the south of the country, from values obtained by BMI, WC and %BF. In order to monitor the morbidities associated with fat accumulation before adulthood, it is vital to create policies that foster health and changes in lifestyle [19].

With regard to nutritional status based on BMI, the overweight population represents 26.7% of the sample (20.9% overweight and 5.8% obese). Studies conducted in other countries portray prevalences of overweight and obesity as follows: 15.6% for Polish adolescents, out of which 10.3% are overweight and 5.3% are obese in a sample of 456 individuals [7]; 35.1% and 20.3% for overweight and obese USA adolescents, respectively [20], and; 14.3% and 2.9% in 5564 Indians of the same age group [21]. In Brazil the percentages for overweight and obesity of 17.8% and 9.8% [22], and 30% and 31.8% [23] can be found. It is also worth noting that according to the last census the values found in this study are close to the national values of 21.7% for overweight and 5.9% for obese [24].

WC average value (71.20±7,65cm) was lower than those reached in studies carried out with Spanish adolescents (76.7 ± 10,4cm) [25], as well as with Polish (75.8±9,1cm) [7], Greek (73.8±9,3cm) [26], and Portuguese (76.5±8,2cm) [27]. Concerning the %BF values, the literature shows samples with lower average values [7, 9] and higher [28, 29] to the percentage of 20.63±5.81% found in this study.

Despite the recognized importance of this assessing measure of central fat in children and adolescents, and the significant correlation of this variable with %BF (r = 0.401, p = 0.002) and fat mass (kg) (r = 0.599, p = 0.000), WC was a discarded variable in every equation model tested. Perhaps its use would show a better adaptation to a female sample, as reported in the study by Lyra et al. [30], who developed an equation predicting body fat in adolescents based on body circumference.

ST utilization has been widely recommended to monitor obesity in children and adolescents due to its high sensitivity [31]. Historically, the subscapularis and triceps measures have been used independently, or in multivariate equations, as good indicators of obesity in adolescents [31–33]. The inclusion of these points as independent variables cooperated to the development of regression models that fulfill the criteria set out in this study, with values of r^2^ = 0.850 when used alone, and r^2^ = 0.935 when added to other variables. In addition, adolescence is a critical phase of constant body development, where approximately 20–25% of the height is acquired [34], may explain this variable presence in estimation models with negative factor for %BF gain.

All equations developed in this study were in conformity with DXA reference method. In addition to their consistency, the models showed a tendency to overestimate %BF values in slimmer individuals and underestimate values for obese ones (Figs 2A, 3A and 4A). As referring to children, authors report that DXA technology compared to more accurate multicompartmental methods tend to overestimate %BF in the obese and underestimate in the slim [11, 29], which can strengthen the equations of this study in future research validation with multicompartmental methods.

The predictive power of the new equations show that SEE are in accordance with the standards established by Lohman [18], who describes values <3% as adequate for estimating body fat. The correlation values, in turn, were similar to those found by Flavel et al. [33], who proposed equations for Australian youths (6–17 years), and higher than those found by Lyra et al. [30].

The Bland and Altman test allows comparison of results of two methods while individually enabling behavioral verification of samples elements. Significant bias was not found in any equation, apart from limits of agreement oscillating between -3.97 e 3.87% (EQ3). Studies comparing different ST equations for the %BF assessment reported LOA with a variation between -7.6 and 12.3% in Brazilian adolescents [30], ±18.4% in Spanish [12] and -11.6 to 5.8 in Pakistani [8].

In validation sample, the SEE values remained <3% [18]. In additional, Bland and Altman analysis showed no significant bias in any model. About LOA, the reduction of the number of variables tends to increase the upper and low limits. The maximum oscillation of the LOA was -4.36 to 4.15 (EQ 3) and minimum was 0.09 to 0.97 (EQ 1).

One limitation of this study was to use as validation around 10% of initial population once a large sample could provide more reliable results. However, in this research, our main objective was presented new equations. Brazil is a continental country with innumerable ethnic variations, therefore the authors believe that a strong validation must be carried out in other regions of the country.

In fact, there is no ideal model for assessing body composition, especially one that meets the criterion of precision, accuracy, low cost, and practicality. However, the creation of ST models is a good option for clinical practice, meeting the criterion listed above. Therefore, for the establishment of treatments related to the diagnosis of nutritional status with the lowest amount of evaluation errors, it is suggested the combination of ST equations with other anthropometric techniques [8–12].

## Conclusion

The innovation in this study lies on the presentation of new regression equations to predict the body fat of Southern Brazilian adolescents, based on a representative and heterogeneous sample. Another strong point is the statistical methodology used, as it secured a model that meets appropriate values of r^2^, SEE, collinear control of variables, waste normality and homoscedasticity model, in addition to the feasibility for everyday use by health professionals. This study highlights a difficulty in diagnosing overweight and obesity from %BF values in Brazilian adolescents. Thus, a method that provides an accurate assessment of this population is important for the public health system since it enables the strategic development of measures to control this epidemic, as recommended by the WHO. Finally, we suggest other cross-validation studies with the equations proposed in this research.

## Supporting information

S1 File Descriptive analysis of the sample. (PDF)

S1 Fig Bland and Altman plot for validation sample–Equation 1. (TIFF)

S2 Fig Bland and Altman plot for validation sample–Equation 2. (TIFF)

S3 Fig Bland and Altman plot for validation sample–Equation 3. (TIFF)
