# Supplementary material for: Body composition and prediction equations using skinfold thickness for body fat percentage in Southern Brazilian adolescents
Source: PLoS One. 2017 Sep 14;12(9):e0184854. doi: 10.1371/journal.pone.0184854 (PMC5599014; doi:10.1371/journal.pone.0184854)
Supplement: S1 File — (PDF) [file pone.0184854.s001.pdf]

S1 File. Descriptive of the sample.

Table 1. Descriptive data (main sample)

Descriptives (Main Sample)									
	Age (y)	N	Mean	Std. Deviation	Std. Error	95% Confidence Interval for Mean Lower Bound	95% Confidence Interval for Mean Upper Bound	Minimum	Maximum
Weight (kg)	12.00	39.00	47.54	13.21	2.12	43.26	51.82	30.50	86.00
	13.00	41.00	52.33	11.38	1.78	48.74	55.92	33.70	84.40
	14.00	80.00	59.22	13.24	1.48	56.28	62.17	29.70	116.00
	15.00	67.00	61.82	8.97	1.10	59.63	64.01	42.40	84.80
	16.00	72.00	63.67	9.44	1.11	61.45	65.89	44.90	104.60
	17.00	75.00	68.78	10.06	1.15	66.49	71.06	47.50	100.60
	Total	374.00	60.48	12.72	0.66	59.24	61.82	29.70	116.00
Height (m)	12.00	39.00	1.52	0.08	0.01	1.49	1.54	1.35	1.70
	13.00	41.00	1.61	0.08	0.01	1.58	1.63	1.47	1.76
	14.00	80.00	1.67	0.08	0.01	1.66	1.69	1.44	1.89
	15.00	67.00	1.71	0.05	0.01	1.70	1.72	1.60	1.83
	16.00	72.00	1.74	0.07	0.01	1.72	1.75	1.60	1.98
	17.00	75.00	1.75	0.06	0.01	1.73	1.76	1.62	1.93
	Total	374.00	1.68	0.10	0.01	1.67	1.69	1.35	1.98
Body Mass Index(kg/m²)	12.00	39.00	20.55	4.95	0.79	18.95	22.15	14.85	37.22
	13.00	41.00	20.04	3.21	0.50	19.03	21.05	15.24	32.56
	14.00	80.00	21.02	3.61	0.40	20.22	21.82	14.32	35.80
	15.00	67.00	21.10	2.49	0.30	20.49	21.71	16.38	28.33
	16.00	72.00	21.10	2.70	0.32	20.47	21.74	15.83	31.93
	17.00	75.00	23.09	6.50	0.31	21.85	23.09	16.44	31.18
	Total	374.00	21.19	4.26	0.17	20.86	21.53	14.32	37.22
Wait Circunference (cm)	12.00	39.00	67.91	9.59	1.58	64.72	71.11	55.20	89.60
	13.00	41.00	68.87	8.55	1.35	66.13	71.60	57.00	99.50
	14.00	80.00	70.08	7.67	0.86	68.38	71.79	55.70	97.00
	15.00	67.00	70.97	5.61	0.69	69.59	72.35	58.90	88.00
	16.00	72.00	71.45	6.11	0.72	70.01	72.89	57.00	90.90
	17.00	75.00	75.24	7.36	0.84	73.58	76.92	62.30	103.60
	Total	374.00	71.20	7.65	0.40	70.45	72.01	55.20	103.60
Body Fat (%)	12.00	39.00	27.69	7.49	1.20	25.27	30.12	16.50	43.40
	13.00	41.00	23.75	5.70	0.89	21.96	25.55	16.00	42.20
	14.00	80.00	20.03	4.85	0.54	18.95	21.11	15.10	40.20
	15.00	67.00	19.33	4.26	0.52	18.29	20.37	13.90	37.40
	16.00	72.00	18.06	4.31	0.51	17.05	19.07	13.60	39.60
	17.00	75.00	19.51	4.82	0.55	18.30	20.49	13.50	35.30
	Total	374.00	20.63	5.81	0.30	20.01	21.19	13.50	43.40
Fat Mass (g)	12.00	39.00	14536.63	6981.06	1180.01	12138.55	16934.70	6039.00	28510.00
	13.00	41.00	13021.67	5562.94	890.78	11218.37	14824.96	6891.00	31050.00
	14.00	80.00	12750.80	6929.70	794.89	11167.30	14334.31	6806.00	47491.00
	15.00	67.00	13243.70	6777.64	847.20	11550.70	14936.71	6958.00	45610.00
	16.00	72.00	12323.25	4707.01	575.05	11175.12	13471.38	6460.00	36196.00
	17.00	75.00	14830.16	8365.19	959.55	12918.63	16741.69	7474.00	55068.00
	Total	374.00	13406.26	6778.30	358.75	12700.73	14111.79	6039.00	55068.00

Table 2. Descriptive data (validation sample)

Descriptives (Validation Sample)

	Age	N	Mean	Std. Deviation	Std. Error	95% Confidence Interval for Mean Lower Bound	95% Confidence Interval for Mean Upper Bound	Minimum	Maximum
Weight (kg)	12.00	4.00	44.15	6.33	3.17	34.07	54.23	35.00	48.40
	13.00	5.00	58.50	8.47	3.79	47.98	69.02	51.60	72.30
	14.00	9.00	60.63	7.44	2.48	54.91	66.35	53.70	78.50
	15.00	8.00	59.54	8.98	3.17	52.03	67.04	51.60	78.30
	16.00	8.00	56.25	4.71	1.67	52.31	60.19	48.90	63.40
	17.00	8.00	66.39	14.86	5.25	53.97	78.81	48.40	91.40
	Total	42.00	58.86	10.50	1.62	55.59	62.13	35.00	91.40
Height (m)	12.00	4.00	1.49	0.04	0.02	1.42	1.56	1.46	1.55
	13.00	5.00	1.68	0.06	0.02	1.61	1.75	1.65	1.78
	14.00	9.00	1.69	0.04	0.01	1.66	1.72	1.63	1.74
	15.00	8.00	1.73	0.05	0.02	1.69	1.77	1.66	1.79
	16.00	8.00	1.71	0.06	0.02	1.66	1.76	1.64	1.79
	17.00	8.00	1.72	0.07	0.02	1.67	1.78	1.62	1.82
	Total	42.00	1.69	0.08	0.01	1.66	1.71	1.46	1.82
Body Mass Index(kg/m²)	12.00	4.00	19.93	2.52	1.26	15.92	23.93	16.40	22.25
	13.00	5.00	20.63	1.79	0.80	18.42	22.85	18.73	22.95
	14.00	9.00	21.22	2.53	0.84	19.28	23.16	18.93	26.69
	15.00	8.00	19.84	2.83	1.00	17.47	22.21	17.25	26.16
	16.00	8.00	19.35	1.89	0.67	17.77	20.93	15.77	22.20
	17.00	8.00	22.17	3.66	1.29	19.11	25.23	18.44	27.59
	Total	42.00	20.59	2.70	0.42	19.75	21.43	15.77	27.59
Wait Circunference (cm)	12.00	4.00	66.20	5.37	2.69	57.65	74.75	59.90	72.90
	13.00	5.00	69.86	3.90	1.74	65.02	74.70	67.00	76.10
	14.00	9.00	72.50	5.91	1.97	67.96	77.04	65.90	86.00
	15.00	8.00	69.83	4.47	1.58	66.09	73.56	64.80	80.00
	16.00	8.00	66.56	5.45	1.93	62.00	71.12	56.50	73.20
	17.00	8.00	75.83	7.85	2.78	69.26	82.39	65.50	86.50
	Total	42.00	70.58	6.39	0.99	68.59	72.57	56.50	86.50
Body Fat (%)	12.00	4.00	31.53	4.00	2.00	25.16	37.89	25.70	34.80
	13.00	5.00	19.78	4.04	1.81	14.76	24.80	15.60	24.90
	14.00	9.00	21.01	7.40	2.47	15.32	26.70	14.10	36.80
	15.00	8.00	17.88	2.17	0.77	16.06	19.69	15.60	22.00
	16.00	8.00	17.89	1.27	0.45	16.82	18.95	15.60	20.00
	17.00	8.00	18.98	4.83	1.71	14.94	23.01	14.20	29.10
	Total	42.00	20.29	5.79	0.89	18.48	22.09	14.10	36.80
Fat Mass (g)	12.00	4.00	14267.00	3740.69	1870.34	8314.73	20219.27	8788.00	16869.00
	13.00	5.00	11614.40	3066.31	1371.29	7807.08	15421.72	7793.00	15245.00
	14.00	9.00	13030.75	5993.61	2119.06	8019.97	18041.53	8520.00	23486.00
	15.00	8.00	11857.38	5624.87	1988.69	7154.87	16559.88	8861.00	25621.00
	16.00	8.00	10547.75	1640.00	579.83	9176.67	11918.83	8684.00	14294.00
	17.00	8.00	15615.00	7033.17	2486.60	9735.12	21494.88	7244.00	26494.00
	Total	42.00	12769.44	5110.57	798.14	11156.34	14382.53	7244.00	26494.00

Table 3. Descriptive data of skinfold thickness (n=374)

Descriptive Skinfold Thickness (N = 374)

Age (years)		TR (mm)	SB (mm)	AB (mm)	SI (mm)	TH (mm)	MA (mm)	CH (mm)	BI (mm)	CF (mm)
12	Mean ± SD	16.36±8.21	15.82±10.96	24.78±17.37	17.72±14.43	24.79±12.08	13.70±9.39	15.74±11.00	10.09±5.85	16.59±8.13
	Median ± IA	13.60±10.0	11.30±14.60	23.10±20.90	12.9 ±16.10	20.90±10.80	11.50±12.90	11.50±17.80	7.60±9.70	14.90±10.30
13	Mean ± SD	12.34±5.81	11.69±7.81	17.80±11.62	12.17±8.47	19.01±9.53	10.45±6.89	11.82±8.04	7.19±4.37	12.55±5.90
	Median ± IA	11.15±5.93	9.15±4.20	15.00±14.73	9.95±9.45	15.85±10.45	9.05±6.83	9.15±7.03	5.45±3.78	11.20±8.83
14	Mean ± SD	10.36±4.62	10.91±6.55	16.015±11.55	10.51±9.03	15.43±7.31	9.25±7.27	8.99±5.89	5.56±2.63	10.10±5.33
	Median ± IA	9.00±4.55	8.80±4.20	12.10±9.75	7.40±4.30	12.80±6.45	6.50±4.35	6.80±3.85	4.60±2.00	9.00±3.30
15	Mean ± SD	9.18±3.90	10.43±4.95	14.83±9.73	10.01±7.28	13.85±6.40	8.08±4.34	8.06±4.32	4.83±1.72	9.41±4.69
	Median ± IA	8.60±4.00	9.10±4.50	11.30±8.40	7.30±4.40	12.20±6.70	6.40±3.20	6.70±3.20	4.30±1.50	8.00±4.70
16	Mean ± SD	8.56±3.76	10.11±4.99	13.11±8.34	8.90±6.79	12.81±7.05	7.59±4.93	7.02±3.70	4.52±1.76	7.98±3.68
	Median ± IA	7.80±3.65	8.70±3.25	10.85±5.78	6.80±4.28	10.75±5.43	6.15±2.65	6.00±2.35	4.10±1.30	7.15±2.68
17	Mean ± SD	9.17±4.63	11.40±6.05	16.07±10.65	9.91±7.32	12.66±6.26	8.50±6.10	7.91±4.92	4.61±1.98	8.43±3.88
	Median ± IA	7.90±4.05	9.50±3.85	12.10±9.60	7.00±4.65	11.10±4.40	6.30±3.60	6.10±3.25	4.10±1.55	7.60±3.25
Total	Mean ± SD	10.40±5.47	11.36±6.84	16.35±11.66	10.92±9.00	15.44±8.61	9.16±6.61	9.23±6.63	5.68±3.43	10.17±5.69
	Median ± IA	8.85±4.90	9.10±4.33	12.00±10.58	7.40±5.40	12.60±8.53	6.60±4.33	6.75±4.33	4.60±2.23	8.50±4.90

Where: triceps (TR); subescapularis (SB); Abdominal (AB); Suprailiac (SI); Thigh (TH); Medium Axillary (MA); Chest (CH); Biceps (BI;) Calf (CF)

Table 4. Pearson correlation with body fat percentage.

Correlations (N = 374)

		Height	WC	TR	SB	AB	SI	TH	MA	CH	BI	CF	% BF (DXA)
**Age**	Pearson Correlation	.671**	,284**	-,346**	-,140**	-,179**	-,206**	-,370**	-,202**	-,319**	-,422**	-,406**	,027
	Sig. (2-tailed)	.000	,000	,000	,007	,000	,000	,000	,000	,000	,000	,000	,609
**Height**	Pearson Correlation	1	,418**	-,228**	-,053	-,051	-,078	-,254**	-,071	-,177**	-,267**	-,254**	,174**
	Sig. (2-tailed)		,000	,000	,303	,328	,133	,000	,172	,001	,000	,000	,001
**WC**	Pearson Correlation		1	,446**	,644**	,625**	,608**	,407**	,607**	,534**	,426**	,380**	,599**
	Sig. (2-tailed)			,000	,000	,000	,000	,000	,000	,000	,000	,000	,000
**TR**	Pearson Correlation			1	,827**	,869**	,859**	,913**	,826**	,871**	,891**	,893**	,582**
	Sig. (2-tailed)				,000	,000	,000	,000	,000	,000	,000	,000	,000
**SB**	Pearson Correlation				1	,896**	,903**	,787**	,889**	,877**	,814**	,742**	,626**
	Sig. (2-tailed)					,000	,000	,000	,000	,000	,000	,000	,000
**AB**	Pearson Correlation					1	,956**	,836**	,906**	,909**	,831**	,802**	,652**
	Sig. (2-tailed)						,000	,000	,000	,000	,000	,000	,000
**SI**	Pearson Correlation						1	,823**	,938**	,908**	,849**	,812**	,627**
	Sig. (2-tailed)							,000	,000	,000	,000	,000	,000
**TH**	Pearson Correlation							1	,794**	,839**	,842**	,904**	,571**
	Sig. (2-tailed)								,000	,000	,000	,000	,000
**MA**	Pearson Correlation								1	,896**	,827**	,761**	,612**
	Sig. (2-tailed)									,000	,000	,000	,000
**CH**	Pearson Correlation									1	,910**	,818**	,560**
	Sig. (2-tailed)										,000	,000	,000
**BI**	Pearson Correlation										1	,847**	,533**
	Sig. (2-tailed)											,000	,000
**CF**	Pearson Correlation											1	,557**
	Sig. (2-tailed)												,000

Where: triceps (TR); subescapularis (SB); Abdominal (AB); Suprailiac (SI); Thigh (TH); Medium Axillary (MA); Chest (CH); Biceps (BI;) Calf (CF); Waist Circunference (WC); Body Fat Percentage (%BF)

Table 5. Paired samples test in validation sample

		Paired Differences					t	df	Sig. (2-tailed)
					95% Confidence Interval of the Difference				
		Mean	Std. Deviation	Std. Error Mean	Lower	Upper			
Pair 1	%BF - Equation 3	.30000	3.25584	.50239	-.71459	1.31459	.597	41	.554
Pair 2	%BF - Equation 2	.40952	3.19602	.49316	-.58643	1.40547	.830	41	.411
Pair 3	%BF - Equation 1	.35714	2.81634	.43457	-.52049	1.23478	.822	41	.416

Paired Samples Statistics

		Mean	N	Std. Deviation	Std. Error Mean
Pair 1	%BF DXA	20.2857	42	5.79350	.89396
	EQ3	19.9857	42	4.90423	.75674
Pair 2	%BF DXA	20.2857	42	5.79350	.89396
	EQ2	19.8762	42	5.15652	.79567
Pair 3	%BF DXA	20.2857	42	5.79350	.89396
	EQ1	19.9286	42	5.04237	.77805

Paired Samples Correlations

		N	Correlation	Sig.
Pair 1	GDXA & EQ3	42	.827	.000
Pair 2	GDXA & EQ2	42	.836	.000
Pair 3	GDXA & EQ1	42	.874	.000
