# Supplementary figures and images for: Body composition and prediction equations using skinfold thickness for body fat percentage in Southern Brazilian adolescents
Source: PLoS One. 2017 Sep 14;12(9):e0184854. doi: 10.1371/journal.pone.0184854 (PMC5599014; doi:10.1371/journal.pone.0184854)

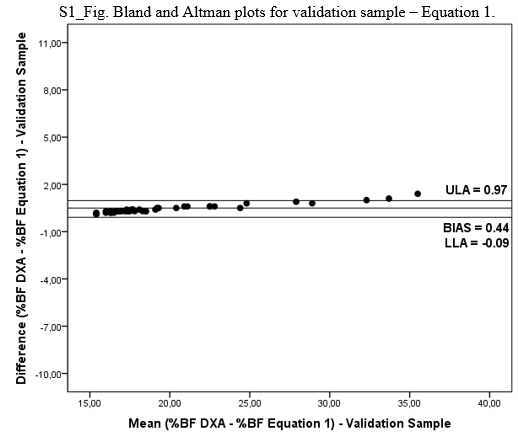

Supplement: S1 Fig — (TIFF) [file pone.0184854.s002.tiff]

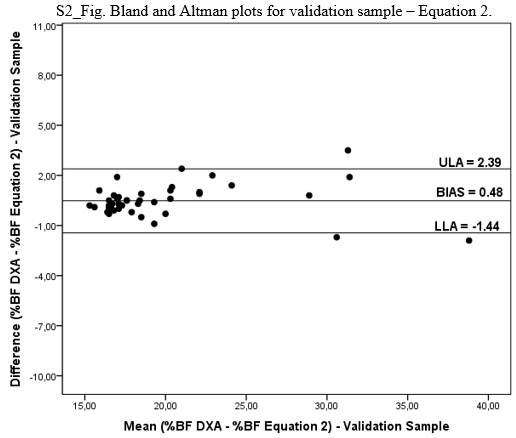

Supplement: S2 Fig — (TIFF) [file pone.0184854.s003.tiff]

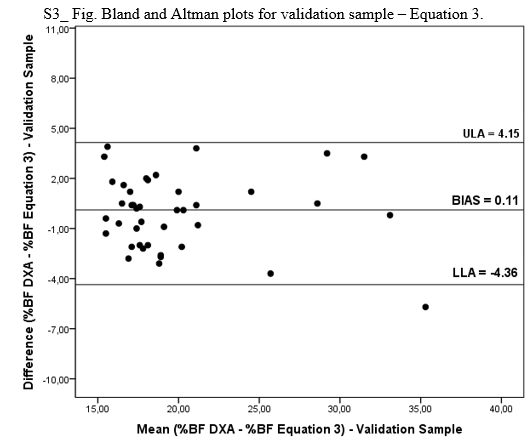

Supplement: S3 Fig — (TIFF) [file pone.0184854.s004.tiff]
